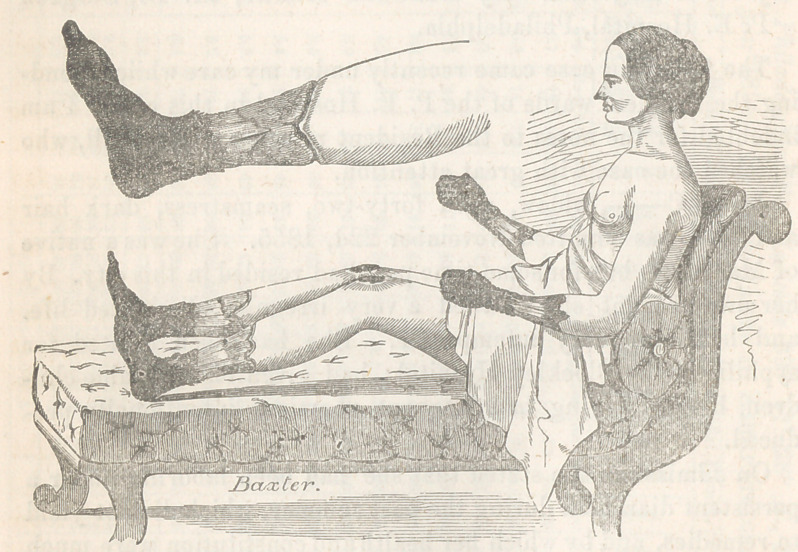# Idiopathic Gangrene of the Four Extremities, Resembling Gangrenous Ergotism

**Published:** 1856-03

**Authors:** Bernard Henry

**Affiliations:** Surgeon P. E. Hospital, Philadelphia


### MEDICAL EXAMINER.

NEW SERIES.—NO. CXXXV.—MARCH, 1856.

### ORIGINAL COMMUNICATIONS.

*Idiopathic Gangrene of the Four Extremities, resembling Gangrenous Ergotism.* By BERNARD HENRY, M. D., Surgeon P. E. Hospital, Philadelphia.

The following case came recently under my care while attending the Surgical wards of the P. E. Hospital in this city. I am indebted for the notes to the Resident physician, Dr. Hall, who watched the case with great attention.

J—— C——, widow, aged forty-two, seamstress, dark hair and eyes, was admitted November 22d, 1855. She was a native of Maryland, but for some time past had resided in this city. By her own account she had led a very irregular, dissipated life, and had been very intemperate. She had been treated for syphilis in the Blockley Hospital; had given birth to nine children, besides having had frequent abortions intentionally produced.

On admission, she stated that she had been laboring under a persistent diarrhœa during the past summer, which did not yield to remedies, and by which her health and constitution were much impaired.

The symptoms of the present disease made their appearance about two weeks previously. November 9th, on returning from the yard, where she had been washing some articles, she felt a stinging sensation in the hands and feet. They were rendered more painful by scratching, and assumed a dusky red color, which became more livid and intense up to the date of her admission into the Hospital.

Dr. West, who had previously seen her, considered that she was laboring under purpura, to which disease her symptoms then bore a strong resemblance.

When she first came under my care, her condition was as follows: the countenance icterode, with an anxious expression, the conjunctivæ yellow, eyelids puffy, the intellect remarkably clear, the hands and forearms, for about a third of their length, of a leaden hue, deepening off to the fingers; these were flexed on the hand, black in color, and dry and shrivelled in appearance. The feet and lower third of the legs were in a similar state. The tip of the nose and the skin over both patellæ were of a dusky color, as though brushed over with bronze paint. The tongue was not much coated, but was marked with two longitudinal reddish-brown stains. The pulse was 80, quick and small.

The affected extremities were icy cold to the touch, and sensibility was so destroyed that the prick of a pin inserted in them was not felt. Sensibility above the line of discoloration was acute.

Movement gave much pain; the weight and warmth of the bed clothes could not be borne; the cold air was more agreeable. The cartilages of the ears showed a commencing similar condition. The bowels at this time were constipated, and the urinary secretion small in quantity.

She was ordered milk punch, opium and nutritious diet. Dr. West had previously prescribed a draught containing a drop of creasote, every four hours. The legs were enveloped in cotton wadding; this was afterwards removed at her own request.

November 24th. The discoloration of the extremities has extended up an inch higher, no line of demarcation is perceptible, the livid hue shading off into the normal color. The pulse remains small, and the urine scanty. Ol. terebinth. gtts. x. every 4th hour were added to the treatment.

November 26th. Vesications, filled with a dark red serous fluid, made their appearance at the edges of the discolored parts. A specimen of urine was obtained passed before breakfast. It was high colored, of a reddish tinge, sp. grav. 1010, reaction alkaline, and exhibits mucus and purpurine.

The case progressed without much alteration in the general symptoms. The lines of demarcation between the sound and affected parts by degrees became more distinct. There was a copious discharge of serous fluid from the vesications. Morphia was given at night to procure sleep, and laxatives to regulate the bowels.

November 30th. Ordered sulph. quin. gr. j; tinct. cinchon. ʒij. every two hours; milk punch, opium, &c. to be continued.

December 3d. The parts are now quite black and dry. The lines of demarcation are distinct, and a slight odor is for the first time perceptible; she sleeps well, and expresses herself as feeling better generally.

Sp. grav. of urine 1010 with an acid reaction. Directed light warm water dressings.

Temperature of different portions of the body to be observed by a thermometer.

December 7th. Pulse in right carotid and brachial artery 92, small and soft. Tongue reddish brown and dry. Temperature under the tongue 102° Fah., in right axilla 96°, in left axilla 100°, right foot and leg 62°, left do. 61°. Temperature of ward, 62°.

During the following 5 or 6 days her condition underwent very little change. The suppuration increased with a most offensive, peculiar odor, unlike that of ordinary gangrene. The limbs were dressed with solution of chloride of soda. It was necessary to increase the doses of the opiates to produce sleep; the urine was secreted in very small quantities, insufficient for examination, and she was ordered to take acetate of potassa, the creasote to be discontinued.

Dec. 11th. Her symptoms more unfavorable, appetite bad; urine still deficient; pulse 80, small. Directed as a diuretic, potass. bitart. grs. iij., potass. iodid., grs. ij., to be substituted for the acetate of potassa. Beef tea to be added to the diet.

Dec. 12th. Complains of burning pain in her stomach; great desire for cold drinks. Vomiting. Tongue dry and brown, passed very little urine, pulse 100°, small. The process of separation more advanced upon the arms than upon the legs, it having extended nearly down to the bone. Upper part of left leg somewhat swollen and infiltrated; changing and removing the dressings causes great pain. From this date to the 15th, no change.

The spirits terebinth. discontinued, owing to the irritability of the stomach.

Dec. 20th. Rather better. Urine still scanty, tongue moist. Takes 2 grs. sulphate morphia at night, ¼ gr. every three hours during the day. Complains much of gagging and disposition to vomit, caused apparently by fœtor. Her appetite continues very bad, with great irritability of the stomach. The line of separation on the arms is complete. The gangrenous portions are dark, dry, shrivelled, resembling an Egyptian mummy, united only by bone and tendon to the sound parts, which show a disposition to granulate.

Dec. 26th. As she appeared rather better than usual to-day, the tongue moist, pulse soft, bowels naturally opened, and urine more abundant, the right hand was removed by sawing through the exposed bones. The granulations were dissected up, to make as fair a stump as possible under the circumstances. No vessels were taken up, but the cut extremities of the bone bled freely. She experienced very little pain, and no inconvenience from the operation. I wished to remove the left hand also, but postponed it at her own request. Directed comp. spts. of lavender, with syrup of ginger and mineral water, to allay thirst and vomiting, with an opiate injection at bed time.

Dec. 28th. Removed the left arm. No disturbance to the patient, the bone, as in the first instance, perfectly sound, and bled freely. Arrested hemorrhage by cold water. No vessels secured.

Dec. 30th. The patient seemed better this morning, having slept soundly for a couple of hours. Tongue pale and clean, pulse small and soft; temperature under the tongue 100°; appetite bad, disposition to vomit.

Very little change took place during the following week; the stumps showed a disposition to heal well, and the line of separation between the sound and gangrenous parts of the lower extremities was so marked as to justify their removal by amputation, did her strength permit.

Her appetite, however, continued bad, she rejected nearly all food, and sleep was procured only by means of large doses of opium.

Tonic and stimulants were administered whenever they could be retained.

On referring to the notes, I find, January 13th, that for the last two days she has been sinking; her mind, which up to the present, has been remarkably clear, begins to fail, she has become irritable and her sensibilities appear blunted. She partially recognized those around her, but could not speak.

Jan. 14th. She remained in a comatose state during the day, and died at 5½ in the evening.

On the first of January, she passed from my care into that of Dr. Wm. Hunt, who took charge of the surgical ward.

*Autopsy thirty hours after Death.*

Present, Doctors West, Hunt, Kenderdine, Hall and Henry.

Emaciation not very great. On opening the thorax and abdomen the viscera were found remarkably dry, scarcely any moisture; very little blood in cutting across the large arterial trunks. The whole venous system appeared engorged with black, thick blood. The lungs were perfectly healthy. Adhesions of the right pleuræ. On opening the pericardium, no fluid was found. The heart was rather small, the coronary veins engorged, as was the whole venous system. The tissue, also, of this organ was more soft than natural, with a tendency to fatty degeneration, and slight fatty deposits in the valves. The pulmonary artery and valves were natural in their structure, but contained a venous clot. The auriculo-ventricular opening was contracted, so as with difficulty to admit the finger. The valves of the aorta were normal ; a coagulum was found in the descending aorta. The brachial and femoral arteries were dissected up and examined ; they presented no unnatural appearance, but were found adherent to the bone, and closed at the line of demarcation.

On opening the abdomen, the liver presented itself fatty and very much enlarged. There appeared to be commencing cirrhosis ; there was resistance to the knife on cutting through the lobuli of that viscus.

The other organs presented nothing remarkable.

The case above recorded presents many points of interest ; it resembles very closely that variety of ergotism accompanied with gangrene of the extremities which has at various times appeared as an epidemic in Europe, principally in Switzerland and in the southern part of France adjoining that country. Very rarely has a single case occurred, it having nearly always made its appearance in the different members of a family, or in the inhabitants of a village or district of country about the same time. Its origin has been traceable to the use of diseased grain ; the harvests have been stinted and the land poor, cold, and subject to inundations where it has made its appearance, or the preceding season very wet. In the patient under our care the circumstances were very different. According to her own account she had always had abundant and excellent food. In this country the harvest of the past year was remarkably good, food is plentiful in our cities, generally of good quality, and within the reach of the poorest classes.

In the July number of the British and Foreign Medico-Chirurgical Review, there is a case reported by Doctor Thos. Camps, which presents a striking resemblance to the one under consideration. The disease appeared chiefly in the lower extremities, which, as he describes, "became black and so shrivelled as to give the idea of nothing intervening between the skin and the bones beneath it." There was the same remarkable aversion to warm covering over the part ; the fingers, the nose and ears were similarly affected. When separation of the gangrenous portions of the limbs commenced, "the fœtor was horribly offensive and peculiar, the soft parts separated to such an extent as to leave a large portion of the bone exposed."

The left leg was removed at this point, close to the granulating surface, and "it was found needful to apply lint to the bone which bled freely." Dr. Camps, quoting from Tissot, states that amputation in the ordinary method has proved fatal in the gangrene of ergotism; but in the epidemic of this disease, which prevailed during the past year in France, in the departments watered by the Loire and Rhone, and which has been noticed in several of the Journals in Europe and of this country, recovery has been known to take place, when performed at the place of election.

In the case under my care, I dissected the granulating surface, and a portion of healthy periosteum, in order to avoid necrosis by exposure, leaving to subsequent circumstances to determine whether to perform amputation in the usual manner. Her recovery, however, was at no time anticipated. Dr. Camps' case got well with the loss of the two lower extremities. This one at one time showed symptoms of amendment, and appeared to resist the ravages of the disease. The nose healed, the sloughs over the patellæ came away, leaving sound healthy surfaces covered with granulations, though rather pale; and the constitutional irritation was comparatively slight. I have been unable to find the record of such extensive and simultaneous gangrene. In nearly all the instances related, but two of the members have been involved, and I am inclined to consider this case as unique.

What was the cause of this condition? The history of the patient shows that she had led a dissolute life, which had undoubtedly impaired the constitution.

Rokitansky says that gangrene arises either from a diseased crasis of the blood, or from a diseased state of the vessels. I am inclined to refer it in this instance to the former cause; though Mr. Barrier, in his account of the epidemic above quoted, states that inspection demonstrated, in nearly all the cases, the existence of primary or secondary arteritis. The autopsy did not reveal to us this fact. The vessels appeared to be sound up to the line of demarcation, where their calibre abruptly closed and they became adherent to the bone and tendons.

The venous congestion, which was remarkable and conspicuous to all present, is similar to what takes place in poisoning. The blood unfitted for nutrition is refused admittance into the capillaries and engorges the venous system. The jaundiced hue of the surface and the petechiæ, indicated the depraved condition of the constituents of that fluid, and the breaking down of the blood corpuscles. The case when first seen by Dr. West, had all the appearance of purpura.

She had eaten rye bread shortly before her attack, but the amount was small; there was no evidence of its being of bad quality, and no other individuals of the family in which she resided, or living in her neighborhood, were simarly affected.

Though she had had frequent miscarriages, it did not appear that ergot had been administered. Abortion, by her own statement, had been produced by Hooper's pills. Had ergot ever been used for such purposes, its effects would have been local, and action transitory. Though so closely resembling the *gangrenous ergotism*, we do not feel justified in classing it in that category; it must, I think, be regarded a case of extensive idiopathic gangrene, occurring in an individual whose constitution was depraved and nutrition impaired by dissipation and debauchery.

The persistence of life under the circumstances of such extensive disease, may be accounted for by the general healthy condition of the principal organs as revealed by dissection. The machinery of life remained comparatively perfect; the nutritive material was diseased. In this view the case bears a resemblance to the form of leprosy, of which I have seen many cases in the Hospital of St. Lazarus, near Bahia. The miserable victims of this disease present the same dried gangrenous condition of the limbs and extremities, with still less evidences of vitality: for the members slough off and separate at the joints without the slightest appearance of granulation or cicatrization, or of any effort on the part of nature to limit the disease.

Unhealthy food, a diet of fish, particularly of the whale, which at certain seasons is caught in the Bay of St. Salvador, and the flesh of which is used for food, is, by the physicians of that country, supposed to give rise to the form of "dry gangrenous leprosy."

Our patient presented a remarkable absence of one of the features accompanying ergotism, as reported by nearly all writers. Her intellect remained clear and unimpaired up to a short period prior to her death: in fact she was particularly intelligent, neither were there observed the spasmodic or convulsive twitchings recorded of that disease.

*Philadelphia, February 19th*, 1856.

## Figures and Tables

**Figure f1:**